# Reply to Peña, A. Comment on “Küppers et al. Percutaneous Anorectoplasty (PARP)—An Adaptable, Minimal-Invasive Technique for Anorectal Malformation Repair. *Children* 2022, *9*, 587”

**DOI:** 10.3390/children9091382

**Published:** 2022-09-14

**Authors:** Julia Küppers, Viviane van Eckert, Nadine R. Muensterer, Anne-Sophie Holler, Stephan Rohleder, Takafumi Kawano, Jan Gödeke, Oliver J. Muensterer

**Affiliations:** 1 Department of Pediatric Surgery, Dr. von Hauner Children’s Hospital, Ludwig-Maximilians-University Medical Center, 80337 Munich, Germany; 2 Department of Pediatric Surgery, Johannes-Gutenberg-University Medical Center Mainz, 55131 Mainz, Germany; 3 Department of Pediatric Surgery, Kagoshima University, Kagoshima 890-8520, Japan

Thank you so much for your thoughtful comments [1] on our recent article “Percutaneous Anorectoplasty (PARP)—An Adaptable, Minimal-Invasive Technique for Anorectal Malformations Repair” [2].

Without a doubt, over the last few decades, PSARP and miniPSARP modifications have revolutionized the care of children with anorectal malformations. Nevertheless, medical progress relies on innovation. Sphincter-sparing techniques such as the laparoscopic-assisted anorectoplasty proposed by Georgeson [3] have been shown to have favorable outcomes concerning long-term functional outcomes in terms of continence [4]. The PARP was devised as a sphincter-sparing technique for the repair of lower anorectal malformations in boys and in those without fistulae in both sexes. It allows real-time image-guided reconstruction for these types of anorectal malformations as well as for internal inspections of the new anal channel and for verifications of a complete and sufficient anocutaneous anastomosis.

Certainly, voluntary continence cannot be evaluated in very young children. However, patients 1, 5, and 7 were indeed fully potty trained at the time of follow-up. Patients 4 and 9 had started potty training and seemed to be able to voluntarily defecate in certain instances. While the other patients were too young to evaluate voluntary continence, they showed no dysfunction for their age, such as permanent smearing or inability of spontaneous defecation. That said, none of the surviving patients required enemas or irrigations, nor did they have other issues with their stooling patterns. Only patient 3 received macrogol therapy for several months because of constipation.

Unfortunately, the patient with Currarino triad died at 6 months due to cardiac reasons, so that continence could not be evaluated. With the exception of this patient, all of the others had normal sacra.

We agree that further studies on the PARP procedure are needed, including careful and long-term assessments of continence, obstipation, and stooling patterns.

Thank you very much for your kind comment on the image of the surgeon taken through the neo-anus from the endoluminal perspective.
